# The Content of Cd and Pb in Herbs and Single-Component Spices Used in Polish Cuisine

**DOI:** 10.1007/s12011-022-03437-7

**Published:** 2022-10-07

**Authors:** Anna Winiarska-Mieczan, Karolina Jachimowicz, Małgorzata Kwiecień, Robert Krusiński, Svitlana Kislova, Lesya Sowińska, Zvenyslava Zasadna, Dmytro Yanovych

**Affiliations:** 1 Institute of Animal Nutrition and Bromatology, University of Life Sciences in Lublin, Akademicka 13, 20-950 Lublin, Poland; 2 State Scientific-Research Control Institute of Veterinary Medical Products and Feed Additives, Lviv, Ukraine

**Keywords:** Herbs, Spices, Cadmium, Lead, Safety

## Abstract

Spices and herbs play an important role in the human diet, mostly due to the presence of essential oils and high antioxidant activity. Therefore, the toxicity of metals raises concerns about the safety of consumption of spices and herbs. This paper examines the content of Cd and Pb in fresh and dried herbs and single-component spices that are the most popular in Polish cuisine: 100 samples of nine kinds of dried herbs, 184 samples of 15 kinds of fresh herbs and 148 samples of 14 kinds of loose single-component spices. The level of Cd and Pb was determined using the GF AAS method. The safety of herbs and spices for consumption was estimated based on the percentage intake of Cd and Pb compared with the tolerable intake (% TWI, % BMDL), chronic daily intake (CDI), target hazard quotient (THQ), hazard index (HI) and comparisons of the results of chemical analyses with the maximum admissible levels of Cd and Pb in Poland and in the European Union. Calculated per fresh weight of the product, dried herbs on average contained 0.134 ± 0.168 mg of Cd and 0.548 ± 0.161 mg of Pb per kg^−1^, fresh herbs on average contained 0.004 ± 0.007 mg of Cd and 0.039 ± 0.033 mg of Pb per kg^−1^, and the mean content of Cd in spices was 0.017 ± 0.019 mg per kg^−1^ and 0.064 ± 0.050 mg of Pb. The % TWI, % BMDL, CDI, THQ and HI (Cd + Pb) for all the analysed products were lower than 1. The results do not imply a risk due to the supply of Cd and Pb with the diet to the human body, primarily due to the small intake of these products. However, it should be highlighted that the content of Cd in dried coriander and estragon and that of Pb in watercress, jiaogulan, celery, basil and dill exceeded the acceptable limit. Thus, their consumption for people from particularly sensitive groups such as babies, pregnant and breastfeeding women and people should be carefully limited.

## Introduction

Spices play an important role in the human diet, mostly due to the presence of essential oils and high antioxidant activity [1, 2, 3]. They improve the taste of food, which allows reducing the use of table salt [4, 5] as its intake is excessive in many countries and is one of the main reasons for the development of cardiovascular diseases. In addition, spices contribute to increasing the intake of vegetables and the preference for low-fat food [6]. Added to fat and stodgy food, they stimulate digestion through boosting gastric juice secretion [7]. They can also alleviate discomfort related to digesting such foods, e.g., passing gas due to fermentation processes or reflux [2, 8]. Some active ingredients of raw spices feature functional qualities such as essential oils that protect the liver and reduce gases, which is most likely connected with their ability to inhibit the activity of 5-lipooxygenase and block α2 adrenergic receptors [9, 10, 11]. The bactericidal and bacteriostatic effects of essential oils present in herbs and spices suppress the development of *Helicobacter pylori*, one of the most significant etiological factors behind peptic ulcers and inflammatory states of the gastric mucosa [11, 12, 13]. At present, the role of herbs and spices and their active ingredients in stimulating metabolic processes is emphasised [14]. The stimulating effect of spices on the gastrointestinal tract is most likely based on two mechanisms: (1) stimulating the liver to secrete bile rich in bile acids necessary for digesting and absorbing fat, and (2) stimulating the activity of digestive enzymes [7].

The fact that how we perceive the taste of food is closely linked to its smell is related to the presence of essential oils. During a short time when food stays in the mouth, olfactory and gustatory para information is transmitted to higher cortical centres of the brain as nervous impulses [15]. These impulses are produced by receptors present in the nasopharynx that receive the para signals, process them and transmit them to higher cortical centres. Studies conducted in Poland show that the most popular cooking herbs are marjoram, oregano and basil [16, 17]. In addition, they reveal that Polish women prefer single-component spices and Polish men like hotter spices with a clearer taste [17].

Unfortunately, herbs and spices, similar to other foodstuffs, can be a source of toxic metals to humans, which is primarily an effect of environmental pollution (air, water and soil), but also of using pesticides and fertilisers for plant growing [18, 19]. Cadmium (Cd) and lead (Pb) are the most common toxic metals in the environment. They have harmful prooxidative, mutagenic, carcinogenic, teratogenic and genotoxic effects [20, 21]. The scale of disorders they cause in the human body is largely dependent on the intake level of such metals. Chronic exposure to Cd and Pb supply is particularly hazardous, as it leads to long-term latent impact on health, while in acute poisoning, an adverse impact is revealed very soon [22, 23]. These metals have a long half-life—from 5 to 30 years for Cd and from 30 days (in soft tissue) to 10 years (in bones) for Pb [24, 25], so their regular supply is dangerous, even in small amounts. Therefore, the consequences of the regular consumption of products containing even small amounts of these metals on human health can be manifested after many years [20]. In 2012, the European Food Safety Authority (EFSA) reduced the tolerable intake level for Cd and Pb. The TWI (tolerable weekly intake) for Cd was determined at the level of 2.5 μg kg^−1^ of body weight per week (0.36 μg kg^−1^ of body weight per day) [26], whereas the BMDL (benchmark dose lower confidence limit) for Pb was: BMDL01 in children—0.5 μg kg^−1^ of body weight per day and BMDL01 in adults—1.5 μg kg^−1^ of body weight per day and BMDL10 in adults—0.63 μg kg^−1^ of body weight per day [27]. No effective methods for reducing the concentration of Cd and Pb in food exist; therefore, humans are constantly exposed to the intake of these metals. Consequently, their content in food should be continually monitored.

Currently, in Poland, there is a tendency to resume a broader outlook on and use herbs and spices as MAPs (medicinal aromatic plants). Spices are officially recommended as an alternative to salt by public health organisations in Poland [28]. People consume spices and herbs for various reasons, the most important of them being a healthy lifestyle. The content of toxic metals in spices depends on multiple factors, including growing and environmental condition, and the species of plants [18, 29, 30]. They can be contaminated with heavy metals during processes associated with their storage and processing [18]. To ensure they are safe to the consumers’ health and lives, agricultural raw materials and products should be strictly monitored for the presence of heavy metals. Therefore, the toxicity of metals raises concerns about the safety of consumption of spices and herbs. This work aimed to examine the content of Cd and Pb in fresh and dried herbs and in single-component spices used in Polish cuisine. It forms part of a project evaluating the intake of toxic and essential minerals by the population in Poland.

## Material and Methods

### Study Material

The content of Cd and Pb was examined in nine kinds of dried herbs (oregano *n* = 15, lovage *n* = 8, rosemary *n* = 9, basil *n* = 16, coriander leaves *n* = 11, coriander seeds *n* = 9, thyme *n* = 15, marjoram *n* = 11, estragon *n* = 7, parsley leaves *n* = 8), in 15 kinds of fresh herbs (oregano *n* = 15, lovage *n* = 9, rosemary *n* = 9, basil *n* = 15, coriander *n* = 10, thyme *n* = 10, marjoram *n* = 10, lemon balm *n* = 11, mint *n* = 15, parsley leaves *n* = 18, sage *n* = 16, jiaogulan *n* = 12, dill *n* = 10, celery leaves *n* = 17, watercress *n* = 10) and 14 kinds of loose single-component spices (turmeric *n* = 12, allspice *n* = 10, white pepper *n* = 9, cayenne pepper *n* = 12, sweet paprika *n* = 10, cumin *n* = 8, ginger *n* = 13, lemon pepper *n* = 10, black pepper *n* = 9, cinnamon *n* = 11, bay leaves *n* = 10, coriander seeds *n* = 15, pink peppercorn *n* = 8, cloves *n* = 11) (Table 1). The products were purchased from groceries in Lublin (eastern Poland) within their shelf-life. All the analysed herbs and spices are used in Polish cuisine and protect or support the gastrointestinal tract [2, 8, 31]. All the fresh herbs used in the study were annuals bought in pots.

**Table 1:** Characteristics of the studied herbs and spices

	Scientific name	n	Origin of the sample	Form	Part used	Therapeutic effect on digestion and digestive system [2, 8, 31]
Herbs						
Oregano	*Origanum vulgare* L	15	Poland, Turkey	Dried	Leaves	Antidiarrheal, antispasmodic, carminative, detoxifying, regulates digestive processes
		12	Poland	Fresh	Leaves and stems	
Lovage	*Levisticum officinale* WDJ Koch	8	Poland	Dried	Leaves	Diuretic, carminative, regulates digestive processes
		9	Poland	Fresh	Leaves and stems	
Rosemary	*Rosmarinus officinalis* L	9	Poland	Dried	Leaves	Disinfectant, choleretic, regulates digestive processes
		9	Poland	Fresh	Leaves and stems	
Basil	*Ocimum basilicum* L	16	Poland, Egypt, Turkey	Dried	Leaves	It soothes digestive disorders, prevents flatulence and stimulates the secretion of gastric juice
		15	Poland	Fresh	Leaves and stems	
Coriander	*Coriandrum sativum* L	11	Poland	Dried	Leaves	Diastolic, carminative, diuretic, stimulating the secretion of gastric acid and appetite
		10	Poland	Fresh	Leaves and stems	
Thyme	*Thymus vulgaris* L	15	Poland	Dried	Leaves	Bacterial, fungicidal and expectorant properties, regulate digestive processes
		10	Poland	Fresh	Leaves and stems	
Marjoram	*Origanum majorana* L	11	Poland, Egypt	Dried	Leaves	Regulates digestion, helps with gastritis and digestive system diseases
		10	Poland	Fresh	Leaves and stems	
Estragon	*Artemisia dracunculus* L	7	Poland	Dried	Leaves	Choleretic, anti-reflux, regulates digestive, fungicidal and antibacterial processes
Savory	*Satureja hortensis* L	8	Poland	Dried	Leaves	Stops excessive fermentation and gas, stimulates digestion
Lemon balm	*Melissa officinalis* L	11	Poland	Fresh	Leaves and stems	Reduces bloating, helps with upset stomach and digestive tract
Mentha	*Mentha* sp*.* L	15	Poland	Fresh	Leaves and stems	Regulates digestive processes, has antibacterial properties
Parsley	*Petroselinum* sp*.* (Mill.) Fuss	18	Poland	Fresh	Leaves and stems	Diuretic, improves digestion
Sage	*Salvia officinalis* L	16	Poland	Fresh	Leaves and stems	Regulates digestive problems, lowers blood sugar levels
Jiaogulan	*Gynostemma pentaphyllum* (Thunb.) Makino	12	Poland	Fresh	Leaves and stems	Antioxidant, anti-inflammatory and anti-stress effects
Dill	*Anethum graveolens* L	10	Poland	Fresh	Leaves and stems	Action: antispasmodic, anti-colic, supports digestive processes
Celery	*Apium graveolens* L	17	Poland	Fresh	Leaves and stems	Regulation of digestive processes, eliminates constipation, diuretic
Watercress	*Nasturtium officinale* WT Aiton	10	Poland	Fresh	Leaves and stems	Prevents constipation, regulates digestive processes, supports liver function
Spices						
Turmeric	*Curcuma longa* L	12	Poland, India	Dried	Rhizomes	Improves liver function, antioxidant, anti-inflammatory, facilitating digestion, choleretic-cholagogue effect
Allspice	*Pimenta dioica* L	10	Poland	Dried	Fruits	Anti-inflammatory qualities, rubefacient effects, comforting the stomach and facilitating digestion
White pepper	*Piper nigrum* L	9	Poland, India	Dried	Fruits	Increases gastric acid secretion, improves digestion and nutrient absorption, increases diuresis, promote proper stomach function, stimulate the release of enzymes, antioxidant, anti-inflammatory
Black pepper	*Piper nigrum* L	12	Poland, Madagascar	Dried	Fruits	
Rose pepper	*Schinus terebinthifolius* G.Raddi	10	Poland, Madagascar	Dried	Fruits	
Lemon pepper	*Capsicum baccatum* L	8	Poland	Dried	Fruits	Anti-inflammatory, anti-diarrhoea and stomach ache
Cayenne pepper	*Capsicum annuum* L	13	Poland, Madagascar, Chine	Dried	Fruits	Influence of gastric emptying, stimulation of gastrointestinal defence and absorption, stimulation of salivary, intestinal, hepatic and pancreatic secretions
Sweet paprika	*Capsicum* sp*.* L	10	Poland	Dried	Fruits	Antioxidant effect, stimulating the gastrointestinal functions, regulate the level of blood sugar
Coriander	*Coriandrum sativum* L	9	Poland, Madagascar	Dried	Seeds	Diastolic, carminative, diuretic, stimulating the secretion of gastric acid and appetite
Cumin	*Cuminum cyminum* L	11	Poland	Dried	Seeds	Antioxidant, anti-inflammatory, antidiabetic effect, pancreatic digestive enzymes secretion, gastroprotective effect
Ginger	*Zingiber officinale* Rosc	10	Poland, Madagascar	Dried	Rhizomes	Anti‐inflammatory, antioxidant, antiulcer, anti-hyperglycaemic effects
Cinnamon	*Cinnamomum* sp*.* J Presl	15	Poland, Zanzibar, Vietnam, Indonesia, Madagascar	Dried	Bark	Anti-inflammatory, antioxidant, antitumor, cardiovascular, cholesterol-lowering, hyperglycaemic effects
Bay leaf	*Cinnamomum tamala* (Buch-Ham) T.Nees & Eberm	8	Poland	Dried	Leaves	Antioxidant, anti-diabetic, diuretic, appetite stimulant
Cloves	*Syzygium aromaticum* L	11	Poland, Madagascar	Dried	Flower buds	Antioxidant, anti-fungal, anti-viral, anti-microbial, anti-diabetic, anti-inflammatory effect

### Preparation of Samples for Analyses

Fresh herbs were dried at 65 °C over 24 h [20, 32], and then ground in a grinder fitted with plastic blades and placed in plastic, tightly sealed containers for up to 5 days. All dried products (dried herbs and spices) were ground in a grinder fitted with plastic blades [29]. The ground samples were placed in plastic, tightly sealed containers for up to 5 days until chemical analyses.

### Analytical Figure of Merits

The material was mixed manually immediately prior to weighing the samples. Samples of ca. 3 g were weighed into China crucibles in two replications per sample. The samples were ash-dried in a muffle furnace at 450 °C for 12 h using H_2_O_2_ as an oxidant. The process was repeated several times until white ash was produced that was later dissolved in 10 ml of 1 M HNO_3_. The methods were described in detail elsewhere [20, 32]. The content of Cd and Pb was determined by GF AAS (graphite furnace atomic absorption spectroscopy) in a Varian Spectr AA 880 apparatus (Table 2). Each analysis was repeated three times, and deviations between the replications did not exceed 5%. The accuracy of determination was verified in a blind test (1 M HNO_3_) using reference materials such as INCT-TL-1 (tea leaves) and INCT-MPH-2 (mixed Polish herbs) containing 0.030 mg of Cd and 1.78 mg of Pb, and 0.100 mg of Cd and 2.16 mg of Pb per 1 kg, respectively. The reference curve was drawn based on standard Cd and Pb solutions from which solutions containing 0.00, 0.10, 0.20, 0.40, 1.00 and 2.00 μg of Cd and of Pb per 1 ml of deionised water were prepared [32].

**Table 2:** Characteristic of parameters for the determination of Pb and Cd by GF AAS

	Cd	Pb
Wave length, nm	228.8	217.0
Lamp current, mA	4	10
Spectral band pass, nm	0.5	1.0
LOD, mg kg^−1^	0.001	0.011
LOQ, mg kg^−1^	0.0002	0.003
Pure gas	Argon	Argon
Background correction	Zeeman	Zeeman
The deviation of duplicate measurement, %	5.8	4.5
Reproducibility, %	10.1	13.9
Quality control		
Blank sample	1 M HNO_3_	1 M HNO_3_
Certified reference materials	INCT-TL-1 (tea leaves)	INCT-TL-1 (tea leaves)
	INCT-MPH-2 (mixed Polish herbs)	INCT-MPH-2 (mixed Polish herbs)
Certified element concentration in INCT-TL-1		
Certified, mg kg^−1^	0.030	1.78
Observed, mg kg^−1^	0.031	1.77
Recovery rate, %	103	99
Certified element concentration in INCT-MPH-2		
Certified, mg kg^−1^	0.199	2.16
Observed, mg kg^−1^	0.187	2.21
Recovery rate, %	94	102

### Chemical Reagents

Hydrogen peroxide H_2_O_2_ and nitric acid HNO_3_ were bought from POCH S.A. (Poland). Standard solutions of Cd and Pb used to draw the reference curve were purchased from Merck (Germany); they contained 1000 mg of Cd (as CdC1_2_) and 1000 mg of Pb (as Pb(NO_3_)_2_) per 1 L of H_2_O. Certified reference materials INCT-TL-1 and INCT-MPH-2 were obtained from the Institute of Nuclear Chemistry and Technology (Warsaw, Poland).

### Calculations and Statistical Analysis

Safety of herbs and spices for consumers was estimated based on several indicators:

- (1) Calculation of the percentage of Cd and Pb intake compared with the acceptable levels proposed by EFSA, that is, tolerable weekly intake (TWI), and benchmark dose lower confidence limit (BMDL) calculated from the following formulas [33]:
  $$\%\mathrm{TWI}=\frac{\mathrm{estimated}\;\mathrm{weekly}\;\mathrm{intake}\;\mathrm{of}\;\mathrm{Cd}\times100}{TWI}$$
  $$\%\mathrm{BMDL}=\frac{\mathrm{estimated}\;\mathrm{weekly}\;\mathrm{intake}\;\mathrm{of}\;\mathrm{Pb}\times100}{\mathrm{BMDL}}$$

The estimated weekly intake (EWI) and the estimated daily intake (EDI) of Cd and of Pb was calculated using the following formulas [33]:

$$\mathrm{EWI}=\frac{\mathrm{mean}\;\mathrm{weekly}\;\mathrm{consumption}\;\mathrm{of}\;\mathrm{herbs}\;\mathrm{or}\;\mathrm{spices}\times\mathrm{Cd}\;\mathrm{or}\;\mathrm{Pb}\;\mathrm{level}}{\mathrm{body}\;\mathrm{weight}}$$

$$\mathrm{EDI}=\frac{\mathrm{mean}\;\mathrm{daily}\;\mathrm{consumption}\;\mathrm{of}\;\mathrm{herbs}\;\mathrm{or}\;\mathrm{spices}\times\mathrm{Cd}\;\mathrm{or}\;\mathrm{Pb}\;\mathrm{level}}{\mathrm{body}\;\mathrm{weight}}$$

The TWI is 2.5 μg of Cd per kg^−1^ of body weight per week [26], BMDL01 is 10.5 μg of Pb per kg^−1^ of body weight per week, and BMDL10 is 4.4 μg of Pb per kg^−1^ of body weight per week [27]. The mean daily intake of herbs in Poland is 0.7 g [18], the mean daily intake of spices in Poland is 0.7 g [18], and the average body weight of adult Poles is 70 kg [33]. Since information on the intake of fresh herbs in Poland is not available, the percentage of TWI and of BMDL and other safety indicators were not calculated for them.

- (2) Exposure to the harmful effects of Cd and Pb consumed with herbs and spices was estimated based on the Chronic Daily Intake (CDI), Target Hazard Quotient (THQ) and the Hazard Index (HI) calculated as below [33, 34, 35, 36]:
  $$\mathrm{CDI}=\frac{\mathrm{EDI}\times\mathrm{EFr}\times{\mathrm{ED}}_{\mathrm{tot}}}{\mathrm{body}\;\mathrm{weight}\times\mathrm{AT}}$$
  $$\mathrm{THQ}= \frac{\mathrm{CDI }}{\mathrm{MPI}}$$
  $$\mathrm{HI}=\mathrm{THQCd}+\mathrm{THQPb}$$

EFr means days of exposure—365 days in a year; ED_tot_ is the duration of exposure—56 years; AT is the mean time of exposure—365 days in a year, MPI (maximum permissible intake) for Cd is 1 µg per kg^−1^ of body weight per day, while for Pb 3.5 µg per kg^−1^ of body weight per day [37]. If THQ > 1, it is likely that potentially harmful effects for human health will occur due to chronic exposure to Cd and Pb [37].

- (3) Comparison of the results of chemical analyses with the maximum permissible levels (MPL) of Cd and Pb in Poland and in the European Union:
  - 0.05 mg of Cd and 0.3 mg of Pb per 1 kg of fresh herbs [38]; 0.2 mg of Cd per 1 kg of fresh herbs [39].
  - 0.3 mg Cd and 2.0 mg Pb per 1 kg of dried herbs and spices containing more than 50% of herbs [40].

The mean values were calculated taking into account three replications for each determination in every sample. Normality of data distribution was tested with Shapiro–Wilk tests. The significance of differences between the mean content of Cd and of Pb in respective kinds of herbs and spices was determined through one-factor analysis of variance (ANOVA) using Duncan’s test. *P* < 0.05 was assumed as a statistically significant level.

## Results

### Content of Cd and Pb in Herbs and Spices

**Fig. 1:**
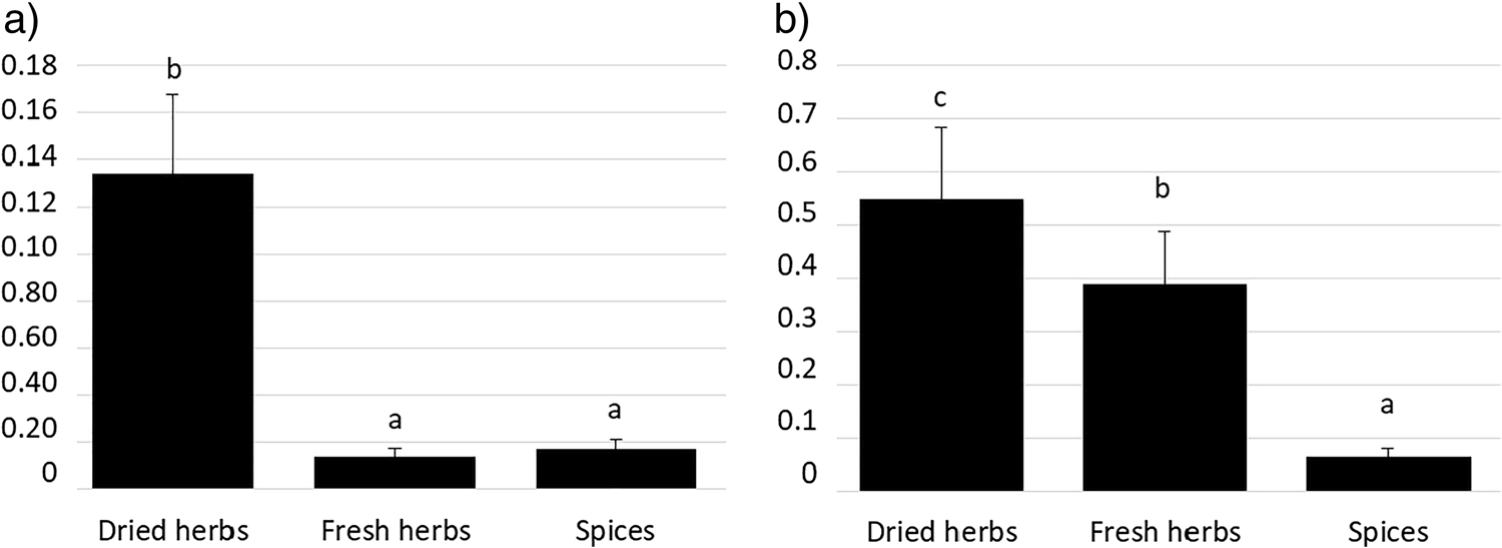
Comparison of Cd (**a**) and Pb (**b**) levels in dried herbs, fresh herbs and spices, mg kg^−1^ dry matter ^a,b,c^Means with different superscripts differ significantly at *P* < 0.05 by Duncan’s test

**Fig. 2:**
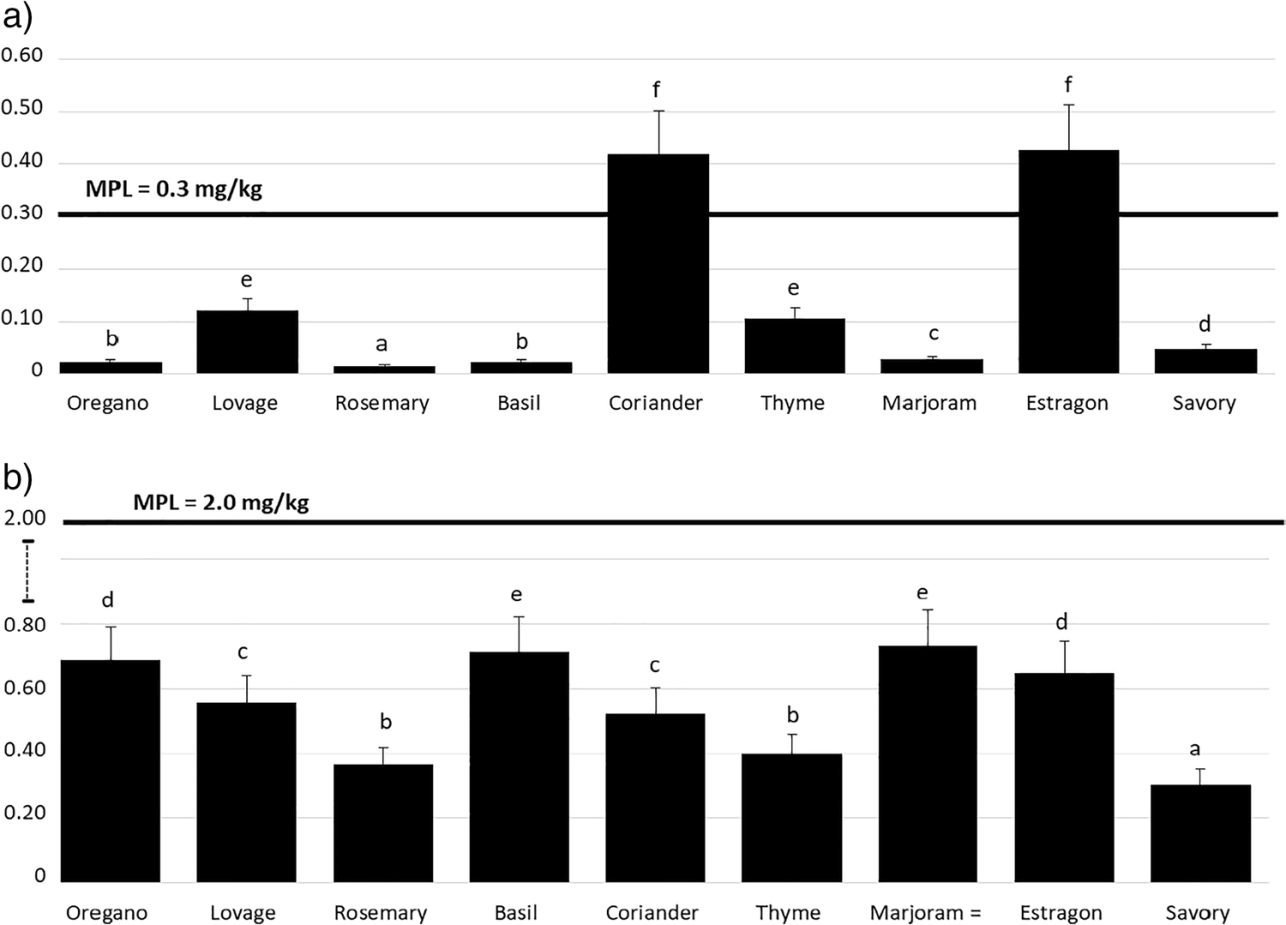
Mean level of Cd (**a**) and Pb (**b**) in analysed dried herbs (leaves and stems), mg kg^−1^ fresh matter; MPL, maximum permissible level [38] ^a,b,c,d,e^Means with different superscripts differ significantly at *P* < 0.05 by Duncan’s test

**Fig. 3:**
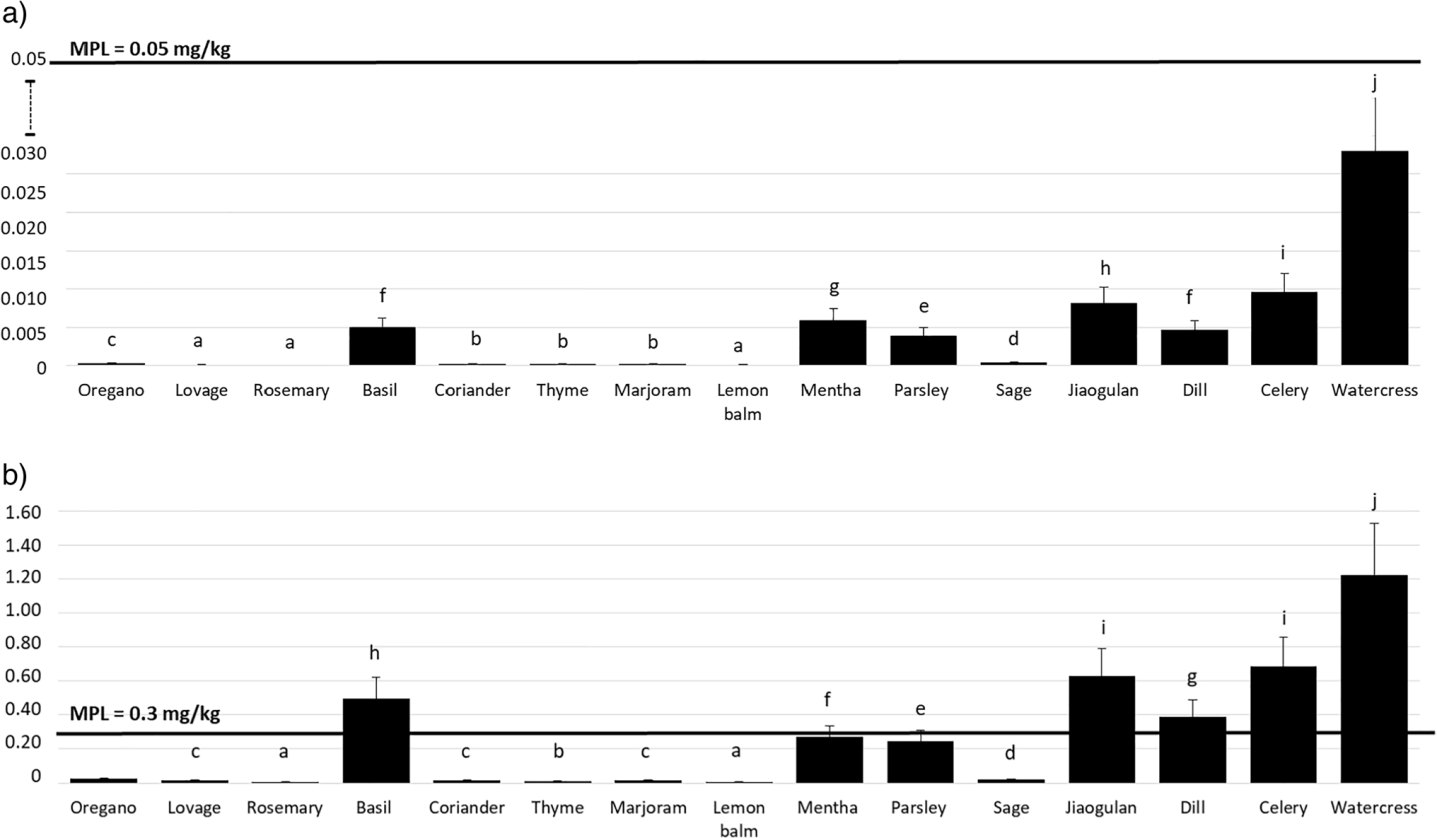
Mean level of Cd (**a**) and Pb (**b**) in analysed fresh herbs, mg kg^−1^ fresh matter; MPL, maximum permissible level [38] ^a,b,c,d,e,f,g,h,i,j^Means with different superscripts differ significantly at *P* < 0.05 by Duncan’s test

**Fig. 4:**
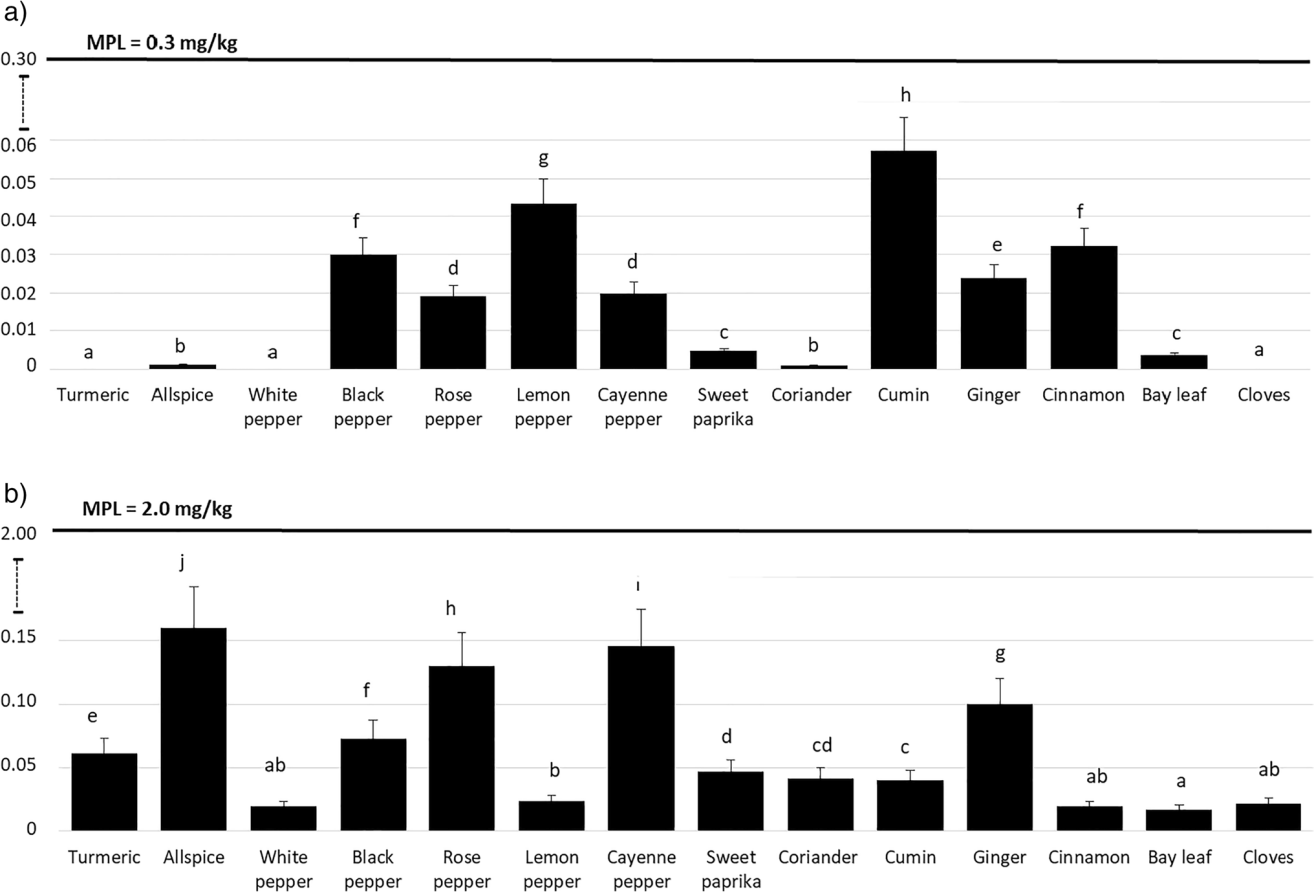
Mean level of Cd (**a**) and Pb (**b**) in analysed spices, mg kg^−1^ fresh matter; MPL, maximum permissible level [38] ^a,b,c,d,e,f,g,h,i,j^Means with different superscripts differs significantly at *P* < 0.05 by Duncan’s test

Calculated per dry weight of the product, the highest (*P* < 0.05) content of Cd was found in dried herbs. No statistically significant differences in the content of Cd in fresh herbs and spices were identified (Fig. 1a). By contrast, calculated per fresh weight of the product, dried herbs on average contained 0.134 ± 0.168 mg of Cd per kg^−1^ (25th percentile 0.047 mg kg^−1^, *n* = 100), fresh herbs on average contained 0.004 ± 0.007 mg of Cd per kg^−1^ (25th percentile 0.002 mg kg^−1^, *n* = 184), and the mean content of Cd in spices was 0.017 ± 0.019 mg per kg^−1^ (25th percentile 0.012 mg kg^−1^, *n* = 148) (Table 3). Depending on the content of Cd, dried herbs can be ordered as follows: coriander = estragon > thyme = lovage > savory > marjoram > oregano = basil > rosemary (Fig. 2a). The order of fresh herbs based on the level of Cd is: watercress > celery > jiaogulan > mint > dill = basil > parsley > sage > oregano > coriander = thyme = marjoram > lemon balm = lovage = rosemary (Fig. 3a). The content of Cd in spices can be represented in the following order: cumin > lemon pepper > black pepper > cinnamon > ginger > rose pepper = cayenne pepper > sweet paprika = bay leaf > allspice = coriander > turmeric = white pepper = cloves (Fig. 4a).

**Table 3:** The content of Cd and Pb in analysed herbs and spices, mg kg^−1^ fresh matter

	Cd	Pb
Dried herbs (*n* = 100)		
Mean	0.134^a^	0.548^b^
Maximum	0.493^a^	0.913^b^
Minimum	0.011^a^	0.248^b^
Median	0.047	0.555
SD	0.168	0.161
Variance analysis	0.028	0.026
Percentile		
75%	0.120	0.688
25%	0.047	0.555
Percent of samples < LOQ	0	0
Fresh herbs (*n* = 184)		
Mean	0.004^a^	0.039^b^
Maximum	0.028^a^	0.122^b^
Minimum	< LOQ	< LOQ
Median	0.002	0.028
SD	0.007	0.033
Variance analysis	0.0000	0.0011
Percentile		
75%	0.005	0.056
25%	0.002	0.028
Percent of samples < LOQ	19	8
Spices (*n* = 148)		
Mean	0.017^a^	0.064^b^
Maximum	0.057^a^	0.160^b^
Minimum	< LOQ^a^	0.017^b^
Median	0.012	0.044
SD	0.019	0.050
Variance analysis	0.0003	0.003
Percentile		
75%	0.028	0.094
25%	0.012	0.044
Percent of samples < LOQ	32	0

Average values for samples, each in three replications; *SD*, standard deviation. ^a,b^Means with different superscripts in the same rows differ significantly at *P* < 0.05 by Duncan’s test. The limit of quantification (LOQ) was 0.0002 mg kg^−1^ for Cd and 0.003 mg kg^−1^ for Pb

Calculated per dry weight of the product, the highest (*P* < 0.05) content of Pb was measured in dried herbs, followed by fresh herbs and spices (Fig. 1b). Calculated per fresh weight of the product, dried herbs contained 0.548 ± 0.161 mg of Pb per kg^−1^ (25th percentile 0.55 mg kg^−1^, *n* = 100), fresh herbs 0.039 ± 0.033 mg of Pb per kg^−1^ (25th percentile 0.028 mg kg^−1^, *n* = 184), and spices on average contained 0.064 ± 0.050 mg of Pb per kg^−1^ (25th percentile 0.044 mg kg^−1^, *n* = 148) (Table 3). In terms of the content of Pb, dried herbs can be ordered as follows: basil = marjoram > oregano = estragon > lovage = coriander > thyme = rosemary > savory (Fig. 2b), while the order of fresh herbs is: watercress > jiaogulan = celery > basil > dill > mint > parsley > sage = oregano > lovage = coriander = marjoram > thyme > rosemary = lemon balm (Fig. 3b). In fresh watercress, jiaogulan, celery, basil and dill, the content of lead was above the MPL, that is, 0.3 mg per kg^−1^ [38]. The order of spices in terms of Pb content is as follows: allspice > cayenne pepper > pink peppercorn > ginger > black pepper > turmeric > sweet paprika > coriander = cumin > white pepper = lemon pepper = cinnamon = cloves > bay leaf (Fig. 4b). The level of Pb in the examined spices was below the MPL.

### Safety of Herbs and Spices for Consumers

Taking into account the mean daily intake of herbs and spices, that is, 0.7 g [18] for the body weight of 70 kg, it was estimated that for dried herbs, the EWI is 6.75E-04 µg of Cd, which corresponds to 3.75E-04% of TWI (Table 4). An intake of 0.7 g of spices is equivalent to 8.33E-05 µg of Cd, that is, 4.76E-05% of TWI. The CDI and THQ for all the analysed products were lower than 1 and amounted to: 9.92E-05 for dried herbs, 3.70E-04 for fresh herbs and 1.26E-05 for spices, respectively (Table 4).

**Table 4:** Safety of culinary herbs and spices for consumption (the results are shown in fresh weight)

	Dried herbs	Fresh herbs	Spices
Cd			
EWI, µg^1^	6.57E-04	2.45E-05	8.33E-05
% TWI^A, B^	3.75E-04	1.40E-05	4.76E-05
CDI^2^	9.92E-05	3.70E-06	1.26E-05
THQ^3^	9.92E-05	3.70E-06	1.26E-05
Pb			
EWI, µg^1^	2.69E-03	1.32E-03	3.14E-04
% BMDL01^A, D^	3.65E-04	1.80E-04	4.27E-05
% BMDL10^A, C^	8.72E-04	4.30E-04	1.02E-04
CDI^2^	4.06E-04	2.00E-04	4.74E-05
THQ^3^	1.16E-04	5.71E-05	1.35E-05
HI^4^	2.15E-04	6.08E-05	2.61E-05

^1^*EWI*, estimated weekly intake calculated on the basis of the mean weekly consumption of culinary herbs and spices and mean level of Cd and Pb; ^2^ *CDI*, chronic daily intake calculated on the basis of the mean weekly consumption of culinary herbs and spices, mean level of Cd and Pb and exposure duration; ^3^*THQ*, target hazard quotient calculated on the basis of the chronic daily intake of Cd or Pb; ^4^ *HI*, hazard index is the sum of THQ for Cd and Pb; ^A^Mean body weight was assumed as 70 kg; ^B^TWI, 2.5 μg Cd per kg of body weight per week [26]; ^C^BMDL01, 10.5 µg Pb per kg of body weight per week [27]; ^D^BMDL10, 4.4 µg Pb per kg of body weight per week[27]

An intake of 0.7 g of dried herbs a day [17] will lead to a weekly intake of 2.69E-03 µg of Pb, which corresponds to 3.65E-04% of BMDL01 and 8.72E-04% of BMDL10 (Table 4). Spices containing a single species of herbs will supply 3.14E-04 µg of Pb weekly, which constitutes 4.27E-05% of BMDL01 and 1.02E-04% of BMDL10. The CDI and THQ for all the analysed products were lower than 1 and amounted to: 4.06E-04 and 1.16E-04 for dried herbs, 2.00E-04 and 5.71E-05 for fresh herbs and 4.74E-05 and 1.35e-05 for spices. In all the analysed cases, HI (Cd + Pb) was lower than 1 and amounted to 2.15E-04 for dried herbs, 6.08E-05 for fresh herbs and 2.61E-05 for single-component spices.

Comparing these results against applicable limits, the level of Cd was found to be excessive only for dried herbs: coriander (mean 0.419 mg kg^−1^) and estragon (mean 0.427 mg kg^−1^), (Fig. 2a). An above-limit level was recorded in two samples of coriander (20%) and in two samples of estragon (30%). The level of Pb was too high compared with the acceptable limit [38, 40] for some fresh herbs only. These included watercress, jiaogulan, celery, basil and dill.

## Discussion

Currently, in Poland, there is a tendency to resume a broader outlook on and use of herbs and spices as medicinal aromatic plants and develop the market for such products. Spices are officially recommended as an alternative to salt by Polish organisations dealing with public health [28]. On the market of spices in Poland and in many other countries, dried spices—accounting for the biggest percentage of turnover—are being replaced with their fresh equivalents [28]. Spices and herbs are consumed for various reasons, particularly as a part of a healthy lifestyle. Therefore, the presence of toxic metals raises concerns about the safety of consumption of spices and herbs.

Polish legislation established separate limits of acceptable content of Cd and Pb for fresh herbs and spices. Thus, fresh herbs can contain a maximum of 0.3 mg of Pb and 0.05 mg of Cd per 1 kg, whereas spices containing more than 50% of herbs—2.0 mg of Pb and 0.3 mg of Cd per 1 kg [38, 40]. The level of cadmium in fresh herbs is also regulated by the legislation of the European Union according to which fresh herbs can contain maximum 0.2 mg of Cd per 1 kg [56].

### Content of Cd and Pb in Herbs and Spices

The concentration of toxic metals in plants depends, among other factors, on their content and bio-availability in soil as well as the species of plant, its morphological part and the length of its vegetation period [29, 30]. The content of heavy metals in the roots of plants was higher than in their aboveground parts [22]. By contrast, the bio-availability of toxic metals in soil, apart from their concentration, is affected by the pH and the oxidation–reduction potential of soil, soil structure and presence of organic matter and endophytes participating in metal translocation to plants [29]. Some plants, including therapeutic ones, show a high phytoremediation potential and have a phytostabilisation and phytoextraction ability [39, 57]. These are normally small slow-growing plants. Tests on therapeutic plants demonstrated that most Cd and Pb accumulates in roots and leaves [58]. It was found that dicotyledons absorb metals much more easily than monocotyledons [59, 60]. Respective parts of plants contain the biggest amount of metals in the following order: root > leaves > stem > flowers > seeds [53, 60]. In the ready raw material, the source of contamination can be technological processes. Contaminants can also derive from auxiliary agents used in the production of food, apparatus, vessels and packaging.

In the presented own study, calculated per dry weight of the product, the content of Cd and Pb was higher (*P* < 0.05) in dried than in fresh herbs. In this study, fresh herbs were annual plants purchased in pots, while dried herbs can include perennial plants. Some researchers demonstrated that the accumulation of heavy metals in plants increases with their age [61], which stems from the capacity of metals to accumulate in living tissue and their long half-life [62]. Furthermore, Cd introduced into soil becomes a part of humus and takes forms that are easily assimilated by plants. In their studies conducted in Poland, Fischer et al. [41] found that commercial samples of herbs contained more Cd than those grown at home. This can be attributed to the fact that chemicals and natural agents used in intensive farming alter soil properties (e.g., reduce its pH) and increase the bio-availability of metals, including toxic metals. Another source of contamination of soil with Cd is phosphorus fertilisers [18, 41] that can be used for industrial and intensive growing of herbs. The growing procedure and data concerning agents used for fertilising and protecting commercial herbs are not publicly available.

In the presented studies, dried coriander and estragon contained more Cd than the acceptable limit (0.3 mg kg^−1^) [38], whereas in spices, this limit did not exceed 0.06 mg kg^−1^. Studies by Gajewska et al. [43] revealed that herbs and spices (*n* = 120; garlic, onion, dill, parsley, basil, oregano, estragon, thyme, curry, turmeric, chilli, nutmeg) available on the retail market in Poland, the mean content of Cd was from 0.011 mg to 0.88 mg kg^−1^ (Table 5). The highest level was measured in Cd. Staniek and Krejpcio [63] found that the level of Cd in the samples of paprika and black pepper available in Poland was 0.015 – 0.017 mg kg^−1^, whereas Fischer et al. [41] observed that the concentration of Cd in herbs grown in Poland ranged from 0.02 to 1.94 mg kg^−1^ and was higher than the maximum limit of Cd in therapeutic plants. Kowalska [18] found that Polish dried herbs and spices (*n* = 224, 12 species of herbs and 11 species of spices) contained from < 0.02 mg to 2.17 mg of Cd per kg^−1^. In the above-mentioned study, the level of Cd in spices was considerably lower than that in herbs. In none of the analysed samples did the level of Cd exceed acceptable limits; the highest mean concentration of Cd was noted in samples of black pepper and cumin seeds—about 0.08 mg per kg^−1^. Reinholds et al. [64] analysed the content of Cd in dried herbs and spices. These authors demonstrated that the level of Cd was the highest in the samples of common thyme (0.4 mg kg^−1^) and basil (0.12 mg kg^−1^), while the lowest levels of this element were recorded in the samples of black pepper (0.01 mg kg^−1^) and marjoram (0.02 mg kg^−1^). Studies conducted in Iran and Iraq showed that samples of herbs and spices contained from 0.012 to 1.64 mg of Cd per kg^−1^ [53, 65, 66]. Turkish researchers observed that the content of Cd in certain herbs was excessive compared with its standard limits, although the evaluation of consumer exposure does not imply a hazard [46]. In turn, studies in Malaysia revealed that the level of Cd in herbs and spices was from 0.23 to 8.07 mg kg^−1^ [67], and those conducted in India found that therapeutic herbs contained from 0.01 to 2.1 mg of Cd per kg^−1^ [68]. Fresh and dried herbs available in the United Arab Emirates (*n* = 81; parsley, sage, basil, oregano, mint, thyme and chamomile) contained from 0.1 to 1.11 mg of Cd per 1 kg [45]. Abualhasan et al. [69] observed that the level of Cd exceeded acceptable limits in all the analysed herbs (*Hibiscus*, anise, chamomile, hawthorn and ginger). By contrast, Baig et al. [55] found that the concentration of Cd in spices (26 species; allspice, areca nut, asafetida, bay leaf, black cardamom, black cumin seed, black pepper, carom seed, cinnamon, cloves, coriander seed, cumin seed, dried fenugreek leaf, dry ginger, fenugreek seed, green, mace, mustard seed, *Nigella* seed, nutmeg, pomegranate seed, red chilli, star anise, tamarind and turmeric) ranged from 0.010 mg to 4.70 mg kg^−1^. The highest concentration of Cd was observed in allspice (3.3–4.7 mg kg^−1^), while the lowest in coriander seeds (0.01–0.02 mg kg^−1^).

**Table 5:** Comparison of the content of Cd and Pb in herbs and spices measured in the present study and reported by other authors, mg kg^−1^ dry matter

Country	*n*	Cd, mg kg^−1^ dry matter	Pb, mg kg^−1^ dry matter	References
Herbs				
Poland	284	< LOQ – 0.43	0.05 – 1.22	This study
Poland	163	< LOQ – 1.67	< LOQ – 5.68	[18]
Poland	48	0.02 – 1.94	No data	[41]
Poland	8	0.04 – 0.78	0.02 – 0.84	[42]
Poland	8	0.01 – 0.88	0.02 – 2.25	[43]
Saudi Arabia	10	< LOD	< LOD – 3.7	[44]
United Arab Emirates	7	< LOD – 1.11	1.44 – 23.52	[45]
Turkey	21	0.32 – 0.52	3.12 – 6.49	[46]
Korea	359	0.01 – 0.31	0.04 – 0.97	[47]
Spices				
Poland	148	< LOQ– 0.06	0.02 – 0.16	This study
Poland	61	< LOQ – 0.082	< LOQ – 1.92	[18]
Poland	4	0.03 – 0.10	0.21 – 0.79	[42]
Romania	7	< LOQ – 0.286	< LOQ – 0.583	[48]
Slovakia	4	0.12 – 1.38	0.04 – 5.59	[49]
Libya	6	0.06 – 0.51	0.56 – 1.06	[50]
Nigeria	5	0.01	0.001	[51]
Zanzibar	15	0.003 – 0.008	0.0004 – 0.001	[52]
Iraq	12	0.012 – 1.32	1.25 – 14.6	[53]
Ethiopia	144	< LOD	< LOD	[54]
Pakistan	26	0.01 – 4.70	0.27 – 52.7	[55]

*LOQ*, limit of quantification; *LOD*, limit of detection

The examined spices and herbs can be considered safe for consumers in terms of the content of Pb that did not exceed the acceptable limit. Gajewska and Czajkowska-Mysłek [42] demonstrated that culinary herbs and spices (garlic, onion, dill, parsley, basil, oregano, estragon, thyme, curry, turmeric, chilli and nutmeg) contained from 0.02 to 0.84 mg of Pb per 1 kg; the highest content of Pb was found in nutmeg and thyme. In the study by Kowalska [18], herbs and spices (*n* = 224) contained from < 0.02 to 5.67 mg of Pb per kg; also, Özden and Özden [46] and Tefera and Teklewoold [54] found that the level of Pb in herbs and spices was consumer-safe. In Malaysian studies, the level of Pb in herbs and spices ranged from 1.54 mg to 8.94 mg kg^−1^ [67], while in Romania, it was on average 0.21 mg of Pb per kg^−1^, whereas the limit was exceeded for one sample which showed 1.42 mg kg^−1^ [48]. Fresh and dried herbs available in the United Arab Emirates (*n* = 81) contained from 1.0 to 23.5 mg of Pb per 1 kg [45]. Studies conducted in Saudi Arabia showed the consumer-safe levels of Pb in spices (turmeric, cloves, black pepper, red pepper, cumin, legume, cinnamon, abazir, white pepper, ginger and coriander) [44]. Harangozo et al. [49] examined the level of Pb in four spices: black pepper, paprika, basil and marjoram available in Slovak shops. The above-named authors found that the spices contained up to ca. 5.6 mg of Pb per kg^−1^; its level was the highest in dried basil—above the acceptable limit. It was observed that herbs purchased from the supermarket contained more Pb than those grown organically [43]. In their studies, herbs from the supermarket contained from 0.03 to 0.96 mg of Pb per 1 kg, and organic herbs from 0.02 to 0.89 mg kg^−1^. Ericson et al. [70] demonstrated that single species of spices and spice blends (*n* = 29; 11 species) available in Georgia showed high concentrations of Pb. The Pb concentration median in six spices exceeded the acceptable limit from 2.4 to 4 times. Studies in Libya showed that among four of the examined spices (chilli, black pepper, turmeric, HRARAT blends containing lesser galangal, ginger and cinnamon), the highest Pb levels were measured in turmeric and chilli— > 1.06 (mean 1.00) mg and 1.02 (mean 0.96) mg per 1 kg, respectively [50]. In studies conducted by Ciotea et al. [71], dried mint (*n* = 4), basil (*n* = 7) and rosemary (*n* = 3) contained from 0.06 to 0.64 mg, from 0.06 to 0.14 mg and from 0.21 to 0.49 mg of Pb per kg^−1^, respectively. Also, Shim et al. [47] observed that herbs and spices (*n* = 359; cinnamon, parsley, basil, oregano, coriander seed, nutmeg, cumin, bay leaf, fenugreek, rosemary, thyme, fennel, sage, clove, marjoram, tarragon, caraway, dill seed, pepper and turmeric) were safe in terms of Pb content, as the level of this metal ranged from 0.039 to 0.972 mg kg^−1^. Makanjuola et al. [51], analysing five kinds of species (onion, nutmeg, garlic, ginger and pepper) found the presence of 0.001 mg of Pb per 1 kg. These values were below the standard limits established by FAO/WHO, so they were considered safe for consumers. The level of Pb amounting to ca. 1 mg per kg^−1^ was measured in cubeb, nutmeg, cinnamon and black pepper consumed in Jordan, irrespective of the origin of imports [72]. In turn, studies conducted in Tanzania (Zanzibar) showed that the level of Pb and that of other heavy metals in cloves is a potential threat to public health; the level of Pb ranged from 0.35 to 1.18 ppm [52]. Ibrahim et al. [53] found that the concentration of heavy metals differed depending on the part of the plant (rhizomes, seeds, leaves and fruits). The above-mentioned authors examined the level of Pb in 12 herbs and spices: black seed, cinnamon, mint, fenugreek, black pepper, ginger, turmeric, dried lime, coriander, sumac fruit, cumin and cardamom. The analysed products contained from 1.25 to 14.6 mg of Pb per 1 kg of dry weight of the product. The highest content was found in fenugreek and cinnamon. These values exceeded standard limits approved by WHO, although the estimation of risk did not show any hazard for consumers when consuming 10 g of spices a day. The level of Pb and other heavy metals in turmeric available in Bangladesh was 12.3 mg kg^−1^, which was higher than acceptable limits. In addition, the concentration of heavy metals was found to be lower in loose packed samples compared with loose unpacked samples [73]. This can imply secondary contamination of the spices as a result of environmental exposure. Studies in Nigeria also revealed that the level of Pb in local spices was too high compared with standard limits and ranged from 2.61 to 8.97 mg kg^−1^ [74]. By contrast, in Pakistan, among 26 analysed kinds of spices, the level of Pb was too high in relation to standard limits in black cumin seeds, areca nuts, carom seeds, green cardamom and pomegranate seeds [55]. In the above-mentioned study, the highest level of Pb (47.3–52.7 mg kg^−1^) was observed in black cumin seeds, and the lowest concentration was measured in ginger (0.265–0.4 mg kg^−1^). Chinese researchers found that among 1773 therapeutic herbs 5.75% exceeded the acceptable content of Pb [29]. It was demonstrated that in therapeutic herbs, the level of Pb is the highest in roots and leaves, and is considerably lower in stems and flowers [75]. About 0.005% of Pb in soil is bio-available to plants. They take up very easily dissolved forms of Pb^2+^ accumulated in roots, and are then transported to the aboveground parts [75]. This is corroborated by the studies of Shim et al. [47] that showed a significantly higher content of Pb in leaf spices than in the fruits and seeds of herbs.

The level of Pb in the presented own studies was exceeded in fresh watercress, jiaogulan, celery, basil and dill, while for dried herbs, the level of Pb was not exceeded; however, the highest (*P* < 0.05) levels of Pb were measured in marjoram and basil. In studies by Kowalska [18], out of 61 samples of dried spices, basil contained the highest amount of Pb. In turn, Dghaim et al. [45] found that basil contained the highest level of Cd (1.11 mg kg^−1^) out of the seven analysed species of herbs (parsley, sage, basil, oregano, mint, thyme and chamomile), similar to the studies by Harangozo et al. [49] where the mean content of Cd in basil exceeded the limit more than two times. In contrast, results obtained by Fischer et al. [41] showed that among the examined species of spices (basil, rosemary, estragon and lovage), basil had the lowest content of Cd (0.2 mg kg^−1^). Studies by Gajewska and Czajkowska-Mysłek [42], on the content of Cd in spices, also demonstrated that the samples of basil contained less Cd than other analysed species of spices. Compared with other herbs, basil is rich in minerals (Fe, Zn and Cu) [76], which can reduce the accumulation of Cd and Pb by this plant.

### Safety of Herbs and Spices for Consumers

Spices and herbs are usually consumed in small amounts in combination with other foodstuffs and do not constitute basic food components. However, depending on the amount consumed, they can be a source of Cd and Pb in the diet. No amount of Cd and Pb exists that can be deemed safe for humans, as these metals are capable of accumulating in tissues and feature a long half-life [24, 25].

In the presented own studies, all the analysed indicators estimating the safety of herbs in terms of the content of Cd and Pb (% TWI, % BMDL, CDI, THQ, and HI) showed very low values, well below 1. Thus, these products can be considered consumer-safe as regards the content of Cd and Pb. Also, Gajewska and Czajkowska-Mysłek [42] demonstrated that culinary herbs are consumer-safe; 3 g of herbs a day will result in an intake of Cd corresponding to ca. 1% of PTWI and that of Pb—to ca. 4% of PTMI. However, it should be highlighted that the above-mentioned authors presumed that the consumption of herbs with the diet was four times higher than in our presented studies that were based on data provided by Kowalska [18], according to which Poles on average consume 0.7 g of herbs and 0.7 g of spices a day. Kowalska [18] found that consumption of herbs and spices increases the risk of THQ for Pb more than for Cd; while, higher values were noted for herbs than for spices. However, it should be underlined that in the above-mentioned studies, these values were lower than 1, which means they are safe for consumers. In Malaysia, the mean consumption of herbs and spices is estimated as 5 g a day, so the exposure to Cd and Pb supplied with these products is 1.11 µg kg^−1^ and 3.21 µg kg^−1^, respectively, corresponding to 15.9% and 12.8% of PTWI [67]. The above-mentioned authors deemed those values to be high, since toxic metals are also consumed with other foodstuffs. Some researches considered spices and herbs unsafe for consumers, as the level of toxic metals present in such products exceeded standard limits [51, 52, 53, 69]. In turn, studies conducted in Nigeria identified a risk associated with the consumption of local African spices*: Prosopis africana, Xylopia aethiopica*, *Piper guineense*, *Monodora myristica* and *Capsicum frutescens* [74]. The above-mentioned authors demonstrated that THQ was lower than 1 (0.06–0.5) and EDI was lower than the tolerable daily intake (TDI); however, the level of Pb exceeded the maximum acceptable concentration about eight to 30 times compared with the standard limit of WHO/FAO, that is, 0.3 mg kg^−1^. In the studies of Baig et al. [55], the content of Cd and Pb in 26 kinds of Pakistan spices was the highest in coriander seed, allspice, *Nigella* seed and black cumin seed, but the estimated consumption of these ingredients fell within WHO’s tolerable weekly intake. Also, the HI was lower than 1, so there was no risk for consumers. 

## Conclusions

The presented results of studies concerning the safety of culinary herbs and spices for consumers in Poland do not imply a risk stemming from the supply of Cd and Pb with the diet to the human body, primarily due to the small intake of these products. However, it should be highlighted that the mean content of Cd in dried coriander and estragon and that of Pb in watercress, jiaogulan, celery, basil and dill exceeded the acceptable limit. Thus, their consumption should be limited for people from particularly sensitive groups such as babies, pregnant and breastfeeding women and people with a weakened immune system. A certain limitation of this study is that people consume different amounts of herbs and spices in their diets. The results obtained represent the actual Cd and Pb content in the products but only estimate the safety of their consumption.

## References

[1] Przeor M, Flarczyk E (2014). The comparison of antioxidant activity of herbs used in Polish cuisine and white mulberry leaves drought. Post Tech Przetw Spoż.

[2] Orkusz A, Bogacz-Radomska L (2017). The importance of spices in human nutrition. Eng Sci Technol.

[3] Bhatti S, Baig JA, Kazi TG, Afridi HI, Pathan AA (2019). Macro and micro mineral composition of Pakistani common spices: a case study. J Food Meas Charact.

[4] Carraro CI, Machado R, Espindola V, Campagnol PCB, Pollonio MAR (2012). The effect of sodium reduction and the use of herbs and spices on the quality and safety of Bologna sausage. Cienc Tecnol Aliment.

[5] Ghawi SK, Rowland I, Methven L (2014). Enhancing consumer liking of low salt tomato soup over repeated exposure by herb and spice seasonings. Appetite.

[6] Polsky S, Beck J, Stark RA, Pan Z, Hill JO, Peters JC (2014). The influence of herbs, spices, and regular sausage and chicken consumption on liking of reduced fat breakfast and lunch items. J Food Sci.

[7] Platel K, Srinivasan K (2004). Digestive stimulant action of spices: a myth or reality?. Indian J Med Res.

[8] Kudełka W, Kosowska A (2008). Components of spices and herbs determining their functional properties and their role in human nutrition and prevention of diseases. Zesz Nauk Uniw Ekonom w Krakowie.

[9] Fifi AC, Axelrod CH, Chakraborty P, Saps M (2018). Herbs and spices in the treatment of functional gastrointestinal disorders: a review of clinical trials. Nutrients.

[10] Embuscado M (2019). Bioactives from culinary spices and herbs: a review. J Food Bioact.

[11] Al Mofleh IA (2010). Spices, herbal xenobiotics and the stomach: friends or foes?. World J Gastroenterol.

[12] Mahady BG, Pendland SL, Stoia A, Hamill FA, Fabricant D, Dietz BM, Chadwick LR (2005). In vitro susceptibility of *Helicobacter pylori* to botanical extracts used traditionally for the treatment of gastrointestinal disorders. Phytother Res.

[13] Nakhaemi MM, Malekzadeh F, Khaje-Karamoddin M, Ramezani M (2006). In vitro anti-Helicobacter pylori effects of sweet basil (*Ocimum basilicum* L.) and purple basil (*Ocimum basilicum* var. purpurascens). Pak J Biol Sci.

[14] Kaufman-Shriqui V, Sherf-Dagan S, Salem H, Navarro DA, Boaz M (2020). Frequently used medicinal herbs and spices in weight management: a review. Funct Foods Health Dis.

[15] Cantone E, Ricciardiello F, Cuofano R, Castagna G, Oliva F, Sequino G, Abate T, Villani R, Iengo M (2017). The human sense of smell. Transl Med Rep.

[16] Żwirska J, Żyła K, Błaszczyk E, Jagielski P, Schlegel-Zawadzka M (2015). Sources of knowledge and application of herbal spices in adults living in the Malopolska and Silesia districts. Bromat Chem Toksykol.

[17] Bortnowska G, Kałużna-Zajączkowska J (2011). Preferences of the powdered spices choice to the meal by professionally working persons in relation to the innovative changes in their manufacturing. Roczn PZH.

[18] Kowalska G (2021). The safety assessment of toxic metals in commonly used herbs, spices, tea, and coffee in Poland. Int J Environ Res Public Health.

[19] Baloch S, Kazi TG, Baig JA, Afridi HI, Arain MB (2020). Occupational exposure of lead and cadmium on adolescent and adult workers of battery recycling and welding workshops: adverse impact on health. Sci Total Environ.

[20] Winiarska-Mieczan A, Grela ER (2017). Content of cadmium and lead in raw, fried and baked commercial frozen fishery products consumed in Poland. J Sci Food Agric.

[21] Arain MB, Kazi TG, Baig JA, Afridi HI, Sarajuddin BKD, Panhwar H, Arain SS (2015). Co-exposure of arsenic and cadmium through drinking water and tobacco smoking: risk assessment on kidney dysfunction. Environ Sci Pollut Res.

[22] Ociepa-Kubicka A, Ociepa E (2012). Toxic effects of heavy metals on plants, animals and humans. Inż Ochr Środ.

[23] Junejo SH, Baig JA, Kazi TG, Afridi HI (2019). Cadmium and lead hazardous impact assessment of pond fish species. Biol Trace Elem Res.

[24] Castelli M, Rossi B, Corsetti FM, Mantovani A, Spera G, Lubrano C, Silvestroni L, Patriarca M, Chiodo F, Menditto A (2005). Levels of cadmium and lead in blood: an application of validated methods in a group of patients with endocrine/metabolic disorders from the Rome area. Microchem J.

[25] Brito JAA, McNeill FE, Webber CE, Chettle DR (2005). Grid search: an innovative method for the estimation of the rates of lead exchange between body compartments. J Environ Monit.

[26] EFSA (2012). Cadmium dietary exposure in the European population. EFSA J.

[27] EFSA (2012). Lead dietary exposure in the European population. EFSA J.

[28] Newerli-Guz J (2017). The analysis of the spices market in Poland. Marketing i Zarządzanie.

[29] Luo L, Wang B, Jiang J, Fitzgerald M, Huang Q, Yu Z, Li H, Zhang J, Wei J, Yang C, Zhang H, Dong L, Chen S (2021). Heavy metal contaminations in herbal medicines: determination, comprehensive risk assessments, and solutions. Front Pharmacol.

[30] Jamnická G, Vál’ka J, Bublinec E (2013). Heavy metal accumulation and distribution in forest understory herb species of Carpathian beech ecosystems. Chem Spec Bioav.

[31] Gawron-Gzella A (2021). Antioxidant activity of popular spices. Post Fitoter.

[32] Winiarska-Mieczan A (2014). Cadmium, lead, copper and zinc in breast milk in Poland. Biol Trace Elem Res.

[33] Baig JA, Chandio IG, Kazi TG, Afridi HI, Akhar K, Junaid M, Naher S, Solangi SA, Malghani NA (2022). Risk assessment of macronutrients and minerals by processed, street, and restaurant traditional Pakistani foods: a case study. Biol Trace Elem Res.

[34] Sultana MS, Rana S, Yamazaki S, Aono T, Yoshida S (2017). Health risk assessment for carcinogenic and noncarcinogenic heavy metal exposures from vegetables and fruits of Bangladesh. Cog Environ Sci.

[35] Issa AB, Yasin K, Loutfy N, Ahmed MT (2018). Risk assessment of heavy metals associated with food onsumption in Egypt: a pilot study. J Clin Exp Tox.

[36] Gruszecka-Kosowska A (2020). Human health risk assessment and potentially harmful element contents in the cereals cultivated on agricultural soils. Int J Environ Res Public Health.

[37] IRIS, Integrated Risk Information System (2015) U.S. Environmental Protection Agency. Chemical assessment summary. National Center for Environmental Assessment. https://cfpub.epa.gov/ncea/iris/iris_documents/documents/subst/0141_summary.pdf (17.04.2022)

[38] Journal of Laws of the Republic of Poland of 13 January 2003 on maximum levels of chemical and biological contaminants which may be present in or on food, food ingredients, permitted additives and processing aids

[39] Khattak MI, Jabeen R, Hameed M (2015). A study of some heavy metals found in medicinal plants (*Euphorbia Cornigera*, *Rhazya Stricta* and *Citrullus Colocynthis*) in Turbat region of Balochistan with reference to prevention of environmental pollution. Pak J Bot.

[40] Journal of Laws of the Republic of Poland of 27 December 2000 on the list of permitted amounts of additives and other foreign substances added to foodstuffs or stimulants, as well as contaminants that may be present in foodstuffs or stimulants

[41] Fischer A, Brodziak-Dopierała B, Steuer M, Rajczykowski K, Kowol J (2017). The concentration of cadmium in spice plants available on the market and in individual growing areas. Environ Med.

[42] Gajewska M, Czajkowska-Mysłek A (2016). The estimation of content of cadmium and lead in culinary herbs and spices available in retail sale. Bromat Chem Toksykol.

[43] Gajewska M, Czajkowska-Mysłek A, Głowacka A (2016). Assesment of content of cadmium and lead in selected dried culinary plants. Post Nauki Technol Przem Rol Spoż.

[44] Seddigi ZS, Kandhro GA, Shah F, Danish E, Soylak M (2016). Assessment of metal contents in spices and herbs from Saudi Arabia. Toxicol Ind Health.

[45] Dghaim R, Al khalib S, Rasool H, Khan MA (2015). Determination of heavy metals concentration in traditional herbs commonly consumed in the United Arab Emirates. J Environ Public Health.

[46] Özden H, Özden S (2018). Levels of heavy metals and ochratoxin a in medicinal plants commercialized in Turkey. Turk J Pharm Sci.

[47] Shim J, Cho T, Leem D, Cho Y, Lee C (2019). Heavy metals in spices commonly consumed in Republic of Korea. Food Addit Contam Part B Surveill.

[48] Voica C, Nechita C, Iordache AM, Roba C, Zgavarogea R, Ionet RE (2021). ICP-MS assessment of essential and toxic trace elements in foodstuffs with different geographic origins available in Romanian supermarkets. Molecules.

[49] Harangozo L, Šnirc M, Árvay J, Bajčan D, Bystrická J, Trebichalský P, Kovarovič J, Jančo I (2018). The heavy metal content in selected kind of spices. J Microbiol Biotechnol Food Sci.

[50] Ziyaina M, Rajab A, Alkhweldi K, Algami W, Al-Toumi O, Rasco B (2014). Lead and cadmium residue determination in spices available in Tripoli City markets (Libya). Afr J Biochem Res.

[51] Makanjuola OM, Osinfade BG (2016). Detection of heavy metals in some seasonings sold in some major highways in Ogun State, South West, Nigeria. Int J Res Stud Biosci.

[52] Khamis FO, Suleiman SA, Sheikh M, Ali AO (2021). Heavy metals content in cloves spices (*Syzygium aromaticum*) cultivated in Zanzibar. Open Access Libr J.

[53] Ibrahim GI, Hassan LM, Baban SO, Fadhil SS (2012). Effect of heavy metal content of some common spices available in local markets in Erbil city on human consumption. Raf J Sci.

[54] Tefera M, Teklewold A (2021). Health risk assessment of heavy metals in selected Ethiopian spices. Heliyon.

[55] Baig JA, Bhatti S, Kazi TG, Afridi HI (2019). Evaluation of arsenic, cadmium, nickel and lead in common spices in Pakistan. Biol Trace Elem Res.

[56] Commission Regulation (EC) No 1881/2006 of 19 December 2006 setting maximum levels for certain contaminants in foodstuffs

[57] Kim HS, Seo BH, Bae JS (2016). An integrated approach to safer plant production on metal contaminated soils using species selection and chemical immobilization. Ecotoxicol Environ Saf.

[58] Łaszewska A, Kowol J, Wiechuła D, Kwapuliński J (2007). Accumulation of metals in selected species of medicinal plants from the area of Beskid Śląski and Beskid Żywiecki. Probl Ekol.

[59] Sauerbeck DR (1991). Plant, element and soil properties governing uptake and availability of heavy metals derived from sewage sludge. Water Air Soil Poll.

[60] Gruca-Królikowska S, Wacławek W (2006). Metals in the environment. Part II. Effect of heavy metals on plants. Chem Dydakt Ekol Metrol.

[61] Czeczot H, Majewska M (2010). Cadmium – exposure and its effects on health. Farm Pol.

[62] Sterckeman T, Thomine S (2020). Mechanisms of cadmium accumulation in plants. Crit Rev Plant Sci.

[63] Staniek K, Krejpcio Z (2013). Evaluation of Cd and Pb content in selected organic and conventional products. Probl Hig Epidemiol.

[64] Reinholds I, Pugajeva I, Bavrins K, Kuckovska G, Bartkevics V (2017). Mycotoxins, pesticides and toxic metals in commercial spices and herbs. Food Addit Contam Part B.

[65] Kohzadi S, Shahmoradi B, Ghaderi E, Loqmani H, Maleki A (2019). Concentration, source, and potential human health risk of heavy metals in the commonly consumed medicinal plants. Biol Trace Elem Res.

[66] Moghaddam M, Mehdizadeh L, Sharifi Z (2020). Macro- and microelement content and health risk assessment of heavy metals in various herbs of Iran. Environ Sci Pollut Res.

[67] Nordin N, Selamat J (2013). Heavy metals in spices and herbs from wholesale markets in Malaysia. Food Addit Contam Part B.

[68] Kumar N, Kulsoom M, Shukla V, Kumar D, Priyanka SP, Kumar S, Tiwari J, Dwivedi N (2018). Profiling of heavy metal and pesticide residues in medicinal plants. Environ Sci Pollut Res.

[69] Abualhasan M, Jaradat N, Sawaftah Z, Mohsen H, Najjar D, Zareer W (2019). Evaluation of heavy metals and microbiological contamination of selected herbals from Palestine. Open Life Sci.

[70] Ericson B, Gabelaia L, Keith J, Kashibadze T, Beraia N, Sturua L, Kazzi Z (2020). Elevated levels of lead (Pb) identified in Georgian spices. Ann Glob Health.

[71] Ciotea D, Ungureanu E, Mustatea G, Popa M (2021). Incidence of lead, cadmium, chromium, nickel and cobalt in basil, rosemary and peppermint seasonings from Romanian market. Bull Univ Agric Sci Vet Med Cluj-Napoca Food Sci Technol.

[72] Al-Dalain SY, Haddad MA, Al-Hawadi JS, Freihat A, Arabiat S, Al-Qudah MMA, Ateyyat MA (2021). Determination of heavy metals and bacterial count of some frequently consumed spices in Jordan. SRP.

[73] Rahman MA, Akter T, Akter R, Sarkar BK, Rafiquzzaman M (2020). Analysis of heavy metal contents in some commercial turmeric samples available at Dhaka, Bangladesh. Jahangirnagar Univ J Biol Sci.

[74] Asomugha RN, Udowelle NA, Offor SJ, Njoku CJ, Ofoma IV, Chukwuogor CC, Orisakwe OE (2016). Heavy metals hazards from Nigerian spices. Rocz Panstw Zakl Hig.

[75] Musielińska R, Śliwińska-Wyrzychowska A (2013). The lead content in selected plant species of potential medicinal significance. Prace Nauk Akad im. Jana Długosza w Częstochowie.

[76] Suliburska K, Kaczmarek J (2011). Evaluation of iron, zinc and copper contents in selected spices available on the Polish market. Roczn PZH.

